# Editorial: Body Representation and Interoceptive Awareness: Cognitive, Affective, and Social Implications

**DOI:** 10.3389/fpsyg.2022.928952

**Published:** 2022-06-08

**Authors:** Simona Raimo, Matteo Martini, Cecilia Guariglia, Gabriella Santangelo, Luigi Trojano, Liana Palermo

**Affiliations:** ^1^Department of Medical and Surgical Sciences, Magna Graecia University of Catanzaro, Catanzaro, Italy; ^2^Department of Psychology, University of East London, London, United Kingdom; ^3^Department of Psychology, “Sapienza” University of Rome, Rome, Italy; ^4^Santa Lucia Foundation IRCCS, Rome, Italy; ^5^Department of Psychology, University of Campania “Luigi Vanvitelli”, Caserta, Italy

**Keywords:** body representation, body self-awareness, bodily self-consciousness, body image, interoception, multiperceptual integration, multisensory processing, heartbeat perception

## Introduction

Following Descartes, we are not in our bodies as pilots are in their vessels (Descartes, 2008). Indeed, when something happens to the body, we actually feel it from within. Most of the scientific interest in understanding how the body is represented in the brain arose from this peculiarity and generated a significant corpus of studies in different research fields.

Converging evidence from body ownership illusion paradigms (e.g., the Rubber Hand Illusion) and patients with peripheral or central nervous system damage strongly suggests that the representation of one's own body arises from the integration of visual, vestibular, tactile, proprioceptive, interoceptive, and motor information (Berlucchi and Aglioti, 2010; Suzuki et al., 2013; Park and Blanke, 2019; Boccia et al., 2020). An efficient body representation (BR) is thought to be central to adequately acting in the environment, constructing the sense-of-self, and interacting with others, with important cognitive, affective and social implications (Goldman and de Vignemont, 2009; Ferroni et al., 2019; Macpherson et al., 2021). BR concerns and disturbances can be determined by disorders of interoceptive processing and multisensory integration, and by implicit and explicit habit-body memory deficits, impacting social and cognitive abilities (Besharati et al., 2016; Badoud and Tsakiris, 2017; Fossataro et al., 2018; Riva, 2018; Raimo et al., 2022).

Several aspects of BR development and dynamics across the lifespan remain to be investigated. Our Research Topic provides a state-of-the-art overview of current investigations, combining experimental psychology, psychiatry, psychophysics, and cognitive and affective neuroscience. In particular, recent BR research is progressively targeting interoceptive processing (i.e., the sense of the physiological condition of the body; Craig, 2002) as an important source of sensory input to bodily cognition. In this vein, the present Research Topic covers the relation between different types of BR and various interoceptive dimensions in motor, cognitive, affective, and social domains.

## Body Representations and Interoceptive Sensibility: Cognitive Implications

Interoceptive sensibility (IS) is a specific component of interoception, corresponding to the self-perceived tendency to focus on interoceptive signals as commonly assessed *via* self-report measures (Garfinkel et al., 2015). In the present Research Topic, four articles focus on IS in healthy individuals investigating (i) the relation with other interoceptive dimensions (i.e., accuracy and awareness) in different modalities (Horváth et al.), (ii) the association with different BR in adult lifespan (Raimo et al.), (iii) sex differences in IS neural correlates (Longarzo et al.), and (iv) IS role in higher-order cognitive functioning (Brown et al.).

Studying the three interoceptive dimensions (i.e., accuracy, sensibility, and awareness) in cardiac and proprioceptive modalities, Horváthet al. suggest that these dimensions are dissociable and modality-specific.

Raimo et al. provide new evidence on the relation between IS and BR, in terms of action-oriented (i.e., body schema) and non-action-oriented (i.e., body structural representation and body semantics) BR, across the adult lifespan. Age-related effects were evident both in action and non-action-oriented BR. Also, higher IS levels were significantly related to worse performance in a task tapping the body schema in older adults.

Along with age, sex differences are another source of interindividual IS variability. Accordingly, Longarzo et al. find both behavioral and neuroanatomical sex differences in IS. Indeed, women reported stronger attention in perceiving inner body sensations. Differences were also found in the strength of the correlation between IS scores and gray matter volumes in specific brain areas, since only in women IS scores and gray matter volumes in the left insula were associated.

A novel contribution to the role of bodily feeling/perception in shaping moral decision-making is offered by Brown et al. Higher IS scores were associated with more intuitive responses and reduced aversion to harmful actions in a task tapping the ability to override intuitive or “gut” responses to counterintuitive problems (i.e., the Cognitive Reflection Task). By implication, this study suggests that higher IS may indicate people's intuitive thinking preference in processing moral dilemma.

## Body Representations and Interoception: Psychopathological and Affective Implications

Our Research Topic provides insight into the role played by BR and interoceptive processing in psychiatric and affective disorders, namely in eating disorders (Khalsa et al.) and depression (Schultchen et al.). Other studies focus on the body-space relation in psychopathologies (Rabellino et al.), and on the interindividual BR variability as a function of schizotypal and autistic traits (Michael et al.; Kuroki et al.) or empathic abilities (Heydrich et al.).

Schultchen et al. find lower interoceptive accuracy in individuals with depression than in healthy controls, a clinically relevant finding since interoceptive abilities can be improved through different methods, including mindfulness.

Khalsa et al. address the safety and tolerability of the Reduced Environmental Stimulation Therapy in anorexia nervosa. This timely paper highlights the need for a more effective form of treatment for anorexia nervosa, and shows the significant effect of this approach in improving interoceptive awareness, and reducing affective symptoms and body image disturbances.

Rabellino et al. provide an overview of the space-body relations, focusing on peripersonal space (PPS). Consistent with a conceptualization of PPS as a protective zone surrounding one's body, authors review studies investigating the relation between PPS, personality traits, and psychopathologies. In particular, they suggest that specific PPS alterations are present in trauma-related disorders.

Michael et al. investigate the relation between the perception of spontaneous sensations, serving to locate the bodily spatial boundaries, and embodiment as seen in schizotypal personality. Higher schizotypal traits were associated with the more frequent perception of spontaneous sensations, signaling altered body boundaries and suggesting that embodiment is required to feel oneself correctly.

Kuroki and Fukui explore how autistic traits modulate performance in hand laterality judgment and self-other discrimination tasks. In men, higher autistic traits were positively correlated with higher reaction times in the hand laterality judgment task. In contrast, in the self-other discrimination task, women with lower autistic trait scores reacted faster to a self-image than to other's images.

Heydrich et al. investigate if adopting another person's perspective can be altered by manipulating interoceptive cues. Participants had to imagine taking the perspective and position of a virtual body presented on a computer screen and to indicate which hand was marked; in the meantime, a silhouette surrounding the virtual body flashed either synchronously or asynchronously with the participants' heartbeats. The effect of synchronous cardio-visual stimulation on visuospatial perspective-taking was only present in participants with high empathic ability, suggesting that interoceptive processing, perspective taking, and empathy are inherently interlinked.

## Bodily Self-Consciousness and Multisensory Integration: Social Implications

Four studies focus on multisensory integration and bodily self-consciousness.

Studying the effect of tactile input on multisensory integration, Tanner, Orthlieb et al. investigate how proprioception interacts with artificial sensory substitution. Participants performed a simple proprioceptive estimation task under four tactile feedback conditions: hover, touch, electrotactile, and vibrotactile. Only the electrotactile and vibrotactile sensory substitutions succeed in multisensory integration when applied to the dominant hand, suggesting that sensory substitution may hinder positional ability in practical application, such as prosthetics.

Tanner, Newman et al. investigate the effect of changing the grip orientation with respect to gravity on the perception of slip direction during active grip, showing that precision grip responses are modulated by task context as seen in forces, latencies, and orientation sensitivity. These findings provide insight for research exploring (own-) body perception and bodily self-awareness and can have implications for future clinical studies in individuals with body modifications (e.g., amputation).

Bekrater-Bodmann et al. explore the relationship between exteroceptive and interoceptive information underlying the feeling that the self is located within the borders of one's own body. By manipulating participants' perspective of their own body (first- vs. third-person perspective) as well as the synchrony of visuotactile stimulation (synchronous vs. asynchronous), the authors found that participants reported out-of-body experiences, particularly under third-person perspective combined with synchronous visuotactile stimulation. Better interoceptive awareness was associated with lower effects of exteroceptive inputs on body perception. Overall, this study nicely shows that bodily self-location, a key component of bodily self-consciousness, relies on the interaction of higher-order interoceptive abilities and exteroceptive input.

Previous research indicated that bodily self-consciousness is also linked to the self-others distinctions, as highlighted in the opinion paper by Pacholik-Żuromska, who discusses how bodily self-consciousness, in terms of sense of ownership and agency, shapes propositional self-others-knowledge (i.e., a specific type of metacognition pivotal in understanding intentions and ascribing mental states to other individuals). Specifically, the author provides a new model of how self-others-knowledge is constructed, assigning a pivotal role to proprioception. This opinion paper calls for more integrative research accounting for the social mind as shaped by the body and its interaction with the world.

## Conclusion

We believe that the collection of articles in this Research Topic underscores the value of combining different perspectives to address body representation and will stimulate further in-depth investigations of such fascinating matter.

## Author Contributions

All authors listed have made a substantial, direct, and intellectual contribution to the work and approved it for publication.

## Conflict of Interest

The authors declare that the research was conducted in the absence of any commercial or financial relationships that could be construed as a potential conflict of interest.

## Publisher's Note

All claims expressed in this article are solely those of the authors and do not necessarily represent those of their affiliated organizations, or those of the publisher, the editors and the reviewers. Any product that may be evaluated in this article, or claim that may be made by its manufacturer, is not guaranteed or endorsed by the publisher.
